# Erratum for Oladeinde et al., “Horizontal Gene Transfer Is the Main Driver of Antimicrobial Resistance in Broiler Chicks Infected with Salmonella enterica Serovar Heidelberg”

**DOI:** 10.1128/mSystems.01147-21

**Published:** 2021-09-28

**Authors:** Adelumola Oladeinde, Zaid Abdo, Maximilian O. Press, Kimberly Cook, Nelson A. Cox, Benjamin Zwirzitz, Reed Woyda, Steven M. Lakin, Jesse C. Thomas, Torey Looft, Douglas E. Cosby, Arthur Hinton, Jean Guard, Eric Line, Michael J. Rothrock, Mark E. Berrang, Kyler Herrington, Gregory Zock, Jodie Plumblee Lawrence, Denice Cudnik, Sandra House, Kimberly Ingram, Leah Lariscy, Martin Wagner, Samuel E. Aggrey, Lilong Chai, Casey Ritz

**Affiliations:** a U.S. National Poultry Research Center, USDA-ARS, Athens, Georgia, USA; b Department of Microbiology, Immunology and Pathology, Colorado State University, Fort Collins, Colorado, USA; c Phase Genomics Inc., Seattle, Washington, USA; d Office of National Programs, USDA-ARS, Beltsville, Maryland, USA; e Institute of Food Safety, Food Technology and Veterinary Public Health, University of Veterinary Medicine, Vienna, Austria; f Austrian Competence Centre for Feed and Food Quality, Safety, and Innovation FFoQSI GmbH, Tulln, Austria; g Division of STD Prevention, National Center for HIV/AIDS, Viral Hepatitis, STD and TB Prevention, Centers for Disease Control and Prevention, Atlanta, Georgia, USA; h National Animal Disease Center, USDA-ARS, Ames, Iowa, USA; i Medical College of Georgia, Augusta, Georgia, USA; j Poultry Science Department, University of Georgia, Athens, Georgia, USA

## ERRATUM

Volume 6, no. 4, e00729-21, 2021, https://doi.org/10.1128/mSystems.00729-21. Page 11: In Table 1, columns headed “Resistance phenotype” and “Antibiotic resistance gene(s),” all instances of “Amp” and “*blaTEM-1B*”, respectively, should not be boldface.

